# Sex Hormone Receptor Signals in Human Malignancies

**DOI:** 10.3390/ijms20112677

**Published:** 2019-05-31

**Authors:** Hiroshi Miyamoto

**Affiliations:** 1 Department of Pathology & Laboratory Medicine, University of Rochester Medical Center, Rochester, NY 14642, USA; 2 Department of Urology, University of Rochester Medical Center, Rochester, NY 14642, USA; 3 James P. Wilmot Cancer Institute, University of Rochester Medical Center, Rochester, NY 14642, USA

Sex steroids, including androgens, estrogens, and progestogens, are known to have widespread physiological actions beyond the reproductive system via binding to the sex hormone receptors, members of the nuclear receptor superfamily that function as ligand-inducible transcription factors. Meanwhile, emerging evidence has indicated the involvement of sex hormone receptor signals in the outgrowth of some malignancies, such as prostate and breast carcinomas, as well as others that have not traditionally been considered as endocrine neoplasms. This Special Issue “Sex Hormone Receptor Signals in Human Malignancies” covers various aspects of the potential role of sex hormone receptors and related signals in prostate cancer [1,2], breast cancer [3,4,5], and other neoplastic conditions [6,7,8,9] by depicting promising findings derived from in vitro and in vivo experiments as well as analyses of surgical specimens.

Capaia et al. [1] investigated the functional role of heterogeneous nuclear ribonucleoprotein K (HNRPK) in androgen-sensitive and castration-resistant prostate cancer cells. Their in vitro data suggested that HNRPK could induce androgen receptor (AR) transactivation and activate downstream targets via functioning as its transcriptional co-regulator. Furthermore, using a co-immunoprecipitation assay coupled with mass spectrometry, they identified several proteins that could interact with HNRPK, as well as AR, and potentially modulated sensitivity to androgen deprivation therapy in prostate cancer. Similarly, Yun et al. [2] assessed the functional role of a BRCA1-interacting protein, COBRA1, in androgen-sensitive and castration-resistant prostate cancer cells. First, COBRA1 expression in prostate cancer was found to correlate with its aggressiveness. In vitro studies then indicated that COBRA1 contributed to promoting cell growth via activating the AR. Moreover, a potent estrogen, 2-methoxyestradiol, was shown to inhibit the growth of even AR-negative DU145 cells, together with down-regulation of COBRA1 expression. These observations may offer potential therapeutic approaches for both androgen-sensitive and castration-resistant prostate cancers via targeting HNRPK and COBRA1.

Forkhead box A1 (FOXA1), as a pioneer factor that modulates the activity of AR and estrogen receptor (ER)-α, has been implicated in the development and progression of prostate and breast cancers [10]. Using high throughput chemical screening and mass spectrometry, Wang et al. [3] identified proteins that could control FOXA1 in breast cancer cells. Of these, cyclin-dependent kinase 1 was suggested to directly regulate FOXA1 via its phosphorylation. Lopez et al. [4] examined the mutational signatures of ER-positive/progesterone receptor (PR)-negative breast cancers and compared the molecular landscapes of PR-negative versus PR-positive tumors. Mutations in the *PIK3CA* (37%) and *TP53* (33%) genes were most frequently seen in PR-negative tumors, with lower (*PIK3CA*: vs. 47%, *p* < 0.01) or higher (*TP53*: vs. 19%, *p* < 0.01) prevalence compared with PR-positive tumors. Additionally, in patients with ER-positive/PR-negative breast cancer, mutations in the *PIK3CA* and/or *TP53* were found to correlate with a significantly worse prognosis. Meanwhile, Hsu et al. [5] summarized available data indicating the involvement of a putative membrane ER, G protein-coupled ER (GPER; also known as GPR30), in breast cancer. Current evidence suggests that GPER plays an important role in mediating the genomic and non-genomic effects of estrogens in breast cancer cells. GPER expression was also suggested to serve as a prognosticator in patients with breast cancer.

Aquino et al. [6] immunohistochemically stained for AR, ERα, ERβ, GPR30, and PR in salivary gland tumor specimens. AR, ERβ, and GPR30 were positive in 25%, 36% (nuclear)/28% (cytoplasmic), and 18% (nuclear)/85% (cytoplasmic) of tumors, respectively, while ERα and PR were negative in all cases examined. In addition, there was a trend to correlate between cytoplasmic ERβ expression and higher grade (*p* = 0.052) or between nuclear GPR30 expression and better disease-free survival (*p* = 0.055). We also used immunohistochemistry to assess the expression status of phospho-ELK1, an activated form of a transcription factor ELK1, in upper urinary tract urothelial carcinoma specimens [7]. Phospho-ELK1 expression was up-regulated in tumors (47.5%; *p* = 0.002), compared with non-neoplastic urothelial tissues (25.3%), and muscle-invasive tumors (54.8%; *p* = 0.065), compared with non-muscle-invasive tumors (35.1%), and was associated with risks of disease progression (*p* = 0.055) and cancer-specific mortality (*p* = 0.008). More interestingly, phospho-ELK1 expression in tumors tended to correlate with AR positivity (*p* = 0.091), especially in male patients (*p* = 0.058). These data support our previous findings in preclinical models [11,12,13] indicating that ELK1 induces urothelial carcinogenesis and cancer growth via cooperation with AR signaling. Another immunohistochemical study by Czogalla et al. [8] determined the expression of ERα and a transcription factor NRF2, which was shown to physically interact with ERα [14], in ovarian cancer tissue samples. The levels of cytoplasmic NRF2 expression were significantly higher in low grade tumors than in high grade tumors (*p* = 0.03). In addition, patients with NRF2-high (*p* = 0.04) or ERα-high (*p* = 0.002) serous cancer showed significantly better overall survival. As expected, inactivation of NRF2 (i.e. cytoplasmic expression in tissues, siRNA expression in cell lines) resulted in up-regulation of ERα protein/mRNA expression, supporting the crosstalk between NRF2 and ERα in ovarian cancer cells. Finally, Coricovac et al. [9] assessed the cytotoxic effects of the components of oral contraceptives in normal skin and skin cancer cells. Ethinylestradiol (10 µM), levonorgestrel (10 µM), or both inhibited the growth of all cell lines examined, especially melanoma cells. However, conflicting results on the effects of contraceptives on the viability of melanoma cells with UVB irradiation were obtained: additional inhibition (in human A375 line) vs. protection against UVB-induced suppression (in murine B164A5 line). Further studies are thus warranted to determine the impact of hormonal therapy with or without irradiation on skin tumorigenesis and tumor progression.

Again, a variety of aspects of the role of sex hormone receptor-mediated signals in human malignancies are described in this Special Issue. The current observations may thus provide a unique insight into novel or known functions of sex hormone receptors and related molecules.
